# Research on High-Performance Underwater-Curing Polymer Composites for Offshore Oil Riser Pipes

**DOI:** 10.3390/polym17131827

**Published:** 2025-06-30

**Authors:** Xuan Zhao, Jun Wan, Xuefeng Qv, Yajun Yu, Huiyan Zhao

**Affiliations:** 1 Chevron China Energy Company, Beijing 100004, China; 2 Shenzhen Branch, CNOOC (China) Co., Ltd., Shenzhen 518067, China; wanjun2@cnooc.com.cn (J.W.); quxf2@cnooc.com.cn (X.Q.); 3 School of Biological and Agricultural Engineering, Jilin University, Changchun 130022, China; huiyan24@mails.jlu.edu.cn

**Keywords:** offshore oil and gas well riser pipes, wellbore integrity, polyisobutylene (PIB), butyl rubber (IIR), solvent-free two-component epoxy system, peel strength

## Abstract

In offshore oil and gas extraction, riser pipes serve as the first isolation barrier for wellbore integrity, playing a crucial role in ensuring operational safety. Protective coatings represent an effective measure for corrosion prevention in riser pipes. To address issues such as electrochemical corrosion and poor adhesion of existing coatings, this study developed an underwater-curing composite material based on a polyisobutylene (PIB) and butyl rubber (IIR) blend system. The material simultaneously exhibits high peel strength, low water absorption, and stability across a wide temperature range. First, the contradiction between material elasticity and strength was overcome through the synergistic effect of medium molecular weight PIB internal plasticization and IIR crosslinking networks. Second, stable peel strength across a wide temperature range (−45 °C to 80 °C) was achieved by utilizing the interfacial effects of nano-fillers. Subsequently, an innovative solvent-free two-component epoxy system was developed, combining medium molecular weight PIB internal plasticization, nano-silica hydrogen bond reinforcement, and latent curing agent regulation. This system achieves rapid surface drying within 30 min underwater and pull-off strength exceeding 3.5 MPa. Through systematic laboratory testing and field application experiments on offshore oil and gas well risers, the material’s fundamental properties and operational performance were determined. Results indicate that the material exhibits a peel strength of 5 N/cm on offshore oil risers, significantly extending the service life of the riser pipes. This research provides theoretical foundation and technical support for improving the efficiency and reliability of repair processes for offshore oil riser pipes.

## 1. Introduction

**Figure 1:**
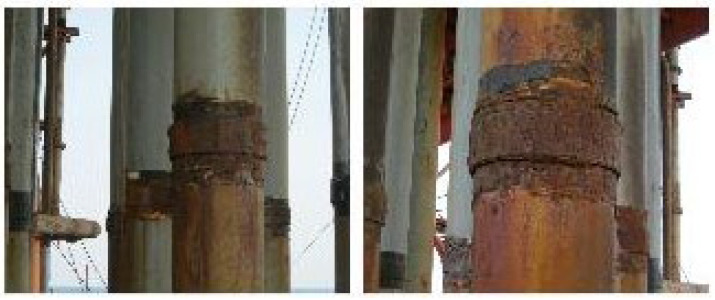
Corrosion conditions at casing joint locations.

With the continuous growth of global energy demand, the development of offshore oil resources has become a core direction in international energy strategy. According to the International Energy Agency (IEA), proven offshore oil and gas reserves account for 34% of total global reserves, and offshore oil production is projected to reach 45% of global total production by 2030 [1,2,3,4]. In offshore oil extraction, riser pipes are critical facilities that ensure wellhead stability and isolate seawater from the wellbore. The casing joint locations have a specialized structure and exist in a rigid connection state. Under the continuous action of periodic impact from offshore waves, joint areas are highly susceptible to fatigue damage [5,6,7], as shown in Figure 1. Data indicate that corrosion-induced failures account for 38% of all offshore engineering equipment accidents annually, with repair costs reaching billions of dollars [8,9,10]. On one hand, complex marine environments accelerate the corrosion process, making it difficult for existing protective measures to provide long-term resistance; on the other hand, the lack of effective remediation measures for already corroded pipes makes riser repair work extremely challenging. As offshore oil development advances into deep and ultra-deep waters, service conditions for riser pipes become increasingly severe, exposing the inadequacies of traditional repair materials and technologies. Therefore, developing high-performance protective materials to extend riser pipe service life, ensure wellbore integrity, and effectively isolate oil and gas has become a critical technical bottleneck in offshore petroleum engineering, with significant implications for production assurance, equipment integrity, cost reduction, and environmental protection.

Traditional riser pipe protection systems primarily rely on metal plating, thermal-sprayed ceramics, and organic coatings. Metal plating [11,12], while cost-effective, is susceptible to galvanic corrosion in seawater. Jin et al. [13] used nickel and iron metals as active anodes and titanium metal as an inert anode. Thermal-sprayed ceramics [14,15,16] (such as alumina) offer excellent high-temperature performance but are highly brittle and complex to apply. Devi et al. [17] synthesized nano-alumina (Al_2_O_3_) particles from waste aluminum foil and evaluated their potential application in corrosion inhibition for low-carbon steel pipelines. Zavareh et al. [18] analyzed the corrosion resistance performance of alumina-titanium (Al_2_O_3_-TiO_2_) oxide ceramic coatings applied on C45 carbon steel pipelines using two different thermal spray processes. Organic coatings [19,20,21,22,23] (such as epoxy resins) are widely used due to their good chemical resistance, but they tend to experience interfacial debonding issues in underwater environments. Li et al. [24] developed a cardanol-based epoxy resin curing agent, selecting cardanol, paraformaldehyde, and m-phenylenediamine as polymer monomers to synthesize the curing agent through Mannich reaction. Zhao et al. [25] provided a novel multi-component amine-cured epoxy resin system, creating elastic, safe, and sustainable materials. Conventional epoxy resin coatings show more than 50% reduction in peel strength after 5 years of service in the South China Sea, failing to meet the 15–20 years design life requirements [26,27]. However, the lack of effective remediation methods for already corroded pipes makes riser repair challenging. In addition to the above problems in the marine protective coatings or repair materials, some scholars have carried out other parametric research and development in the application [28,29,30,31], but for the traditional epoxy coatings in long-term service after peeling strength will be greatly reduced, and some commercial repair materials are usually more than 0.1% of the water absorption rate.

In recent years, polymer composites have become a research focus due to advantages such as strong designability and convenient application. Polyisobutylene (PIB) [32] and butyl rubber (IIR) [33] blend systems show potential in pipeline corrosion prevention due to their low gas permeability, high elasticity, and weather resistance. However, existing research indicates that single PIB/IIR systems face challenges in balancing mechanical properties with adhesive performance. For example, increasing PIB content can enhance material ductility but reduces fixed elongation stress, while the high crosslinking density of IIR limits material processing flowability. Additionally, the performance stability of existing materials across a wide temperature range (−40 °C to 120 °C) and their ability to cure rapidly underwater still require further optimization.

Addressing the corrosion protection challenges of riser pipes in offshore oil and gas extraction, this research utilizes a polyisobutylene (PIB) and butyl rubber (IIR) blend system as a foundation. Through molecular design and multi-scale structure regulation, it systematically resolves technical bottlenecks in mechanical properties, temperature adaptability, and underwater application faced by traditional protective materials. By constructing synergistic reinforcement networks through PIB/IIR molecular chain segment matching, the research overcomes the contradiction between material elasticity and strength. Utilizing the interfacial effects of nano-fillers achieves stable peel strength ≥0.3 N/cm across a wide temperature range from −45 °C to 80 °C. The innovative solvent-free two-component epoxy curing system achieves rapid surface drying and interfacial chemical bonding within 30 min underwater. Through key technologies such as medium molecular weight PIB internal plasticization, nano-silica hydrogen bond reinforcement, and latent curing agent regulation, a novel composite material has been successfully developed with high peel strength (3.8 N/cm), low water absorption (≤0.03%), and stability across a wide temperature range. This research not only reveals the regulatory patterns of PIB content on the viscoelasticity of blend systems but also constructs a theoretical model for underwater-curing material interfacial adhesion, providing a new paradigm for marine engineering protective material design.

## 2. Materials and Methods

### 2.1. Polymeric Internal Protection Materials

**Figure 2:**
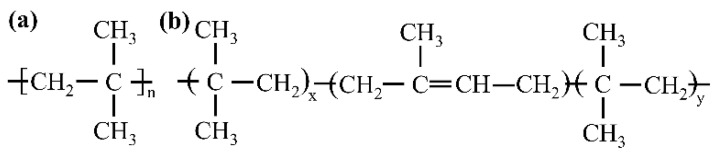
Polymeric protective repair materials. (**a**) Polyisobutylene structure and (**b**) Butyl rubber structure.

Polyisobutylene (PIB, Figure 2a), as a typical linear saturated polymer, exhibits a long-chain structure with an extremely low crosslinking degree. Strong intermolecular forces provide it with excellent substrate adhesion, making it an ideal base material for high-performance adhesives. Butyl rubber (IIR, Figure 2b) is a linear polymer formed by the copolymerization of isobutylene with a small amount of isoprene, which builds a three-dimensional network structure after vulcanization crosslinking. This dense molecular architecture provides excellent chemical stability, thermal stability, and water/gas barrier properties, effectively shielding against corrosive media such as seawater and marine organism secretions. Meanwhile, its low double bond content and high-density side methyl group distribution characteristics provide both high elasticity and impact energy absorption properties, offering elastic constraint protection to the substrate under dynamic loading.

Medium molecular weight polyisobutylene (m-PIB) exhibits a dual role in the blend system. On one hand, it provides an internal plasticization effect through chain segment sliding, a well-established mechanism in compatible polymer blends that enhances flexibility and reduces stiffness [34]. On the other hand, with molecular chain segments significantly longer than low molecular weight PIB, it forms physical entanglement networks with IIR molecular chains [35]. These entanglements act as transient physical crosslinks, achieving reinforcement and toughening of the blended material [36]. Based on its complete compatibility with butyl rubber [37], the m-PIB/IIR blend system can significantly improve the material’s tensile strength, elongation at break, and interfacial adhesion properties. To further optimize the material’s mechanical properties and processing flowability, functional fillers were introduced into the system, along with additives such as dioctyl phthalate (DOP) plasticizer, 2,6-di-tert-butyl-4-methylphenol (BHT) antioxidant, and 2-phenoxyethanol.

### 2.2. Preparation Process of Polymeric Internal Protection Materials

**Figure 3:**
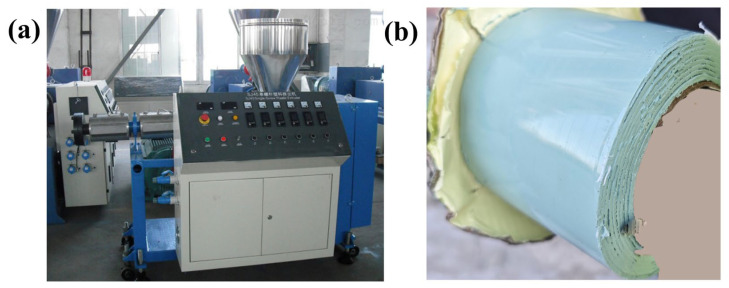
Preparation process. (**a**) Single-screw extruder and (**b**) Prepared composite samples with fiber-reinforced mesh sandwiched between them.

Polyisobutylene, butyl rubber, functional additives, and functional fillers were added to a kneader in different proportions, kneaded at 120 °C for 0.5–1 h, and then compounded through a single-screw extruder at 110–115 °C, as shown in Figure 3a. The mixture was extruded into thin sheets through a 1 mm flat slit die, with fiber-reinforced mesh cloth placed between two prepared thin sheets, as shown in Figure 3b. After cooling, polymeric internal protection material samples with different formulations (labeled as 1#, 2#, 3#, 4#, 5#) were obtained, as shown in Table 1.

### 2.3. Material Characterization and Verification Experiments

#### 2.3.1. Basic Performance Experiments of Polymeric Internal Protection Materials

**Figure 4:**
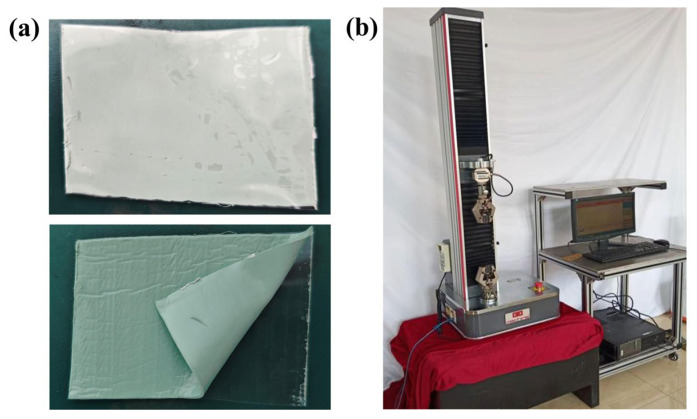
Basic performance experiments. (**a**) Initial adhesion test and (**b**) universal testing machine.

To ensure underwater adhesion performance, initial tack tests (GB/T4852-2002) [38] were first conducted on each sample. At room temperature, the compound was pressed onto a sheet of glass paper (40 × 70 mm) using a flat vulcanizing press. The other side of the compound was covered with polytetrafluoroethylene paper, using a mold to maintain a thickness of 1 mm. The material was then heat-treated in an oven according to vulcanization time data obtained from the vulcanization curve to develop a crosslinked structure. After removing the polytetrafluoroethylene paper, the initial tack of the compound was tested, as shown in Figure 4a.

Additionally, to understand the mechanical properties of each formulation, a universal testing machine was used to determine tensile strength, modulus at specified elongation, and elongation at break for different samples, as shown in Figure 4b.

#### 2.3.2. Repair Testing of Polymeric Internal Protection Materials

After determining the basic properties, repair tests were conducted on the best-performing composite materials, including saturated water absorption rate tests. Two pieces of polymer material were adhesively pressed together and cut into 50 mm × 50 mm specimens (with a thickness of two adhesive tape layers). Three specimens with clean, flat surfaces free of bubbles and cracks were prepared for each group, pre-treated by drying at 50 °C ± 2 °C for 24 h, and then weighed to determine the initial mass m_1_ (with 0.1 mg precision). The specimens were vertically immersed in 25 °C ± 2 °C distilled water, ensuring that the surfaces were free of bubbles and that the specimens did not touch each other. After 24 h of immersion, the specimens were removed, surface water was absorbed with filter paper, and the mass after water absorption m_2_ was immediately weighed. The specimens were then dried again for 24 h and weighed to determine the mass after drying m_3_. The mass increase rate of specimens after water immersion W_pc1_ was calculated according to Formula (1):

W_pc1_ (%) = [(m_2_ − m_1_)/m_1_] × 100% (1)

The mass loss rate of soluble substances in specimens after water immersion S was calculated according to Formula (2):

S (%) = [(m_1_ − m_3_)/m_1_] × 100% (2)

The water absorption rate of specimens W_pc_ was calculated according to Formula (3):

W_pc1_ (%) = W_pc1_ + S (3)

The experimental results were represented as the arithmetic mean of the calculated results for each group of specimens.

**Figure 5:**
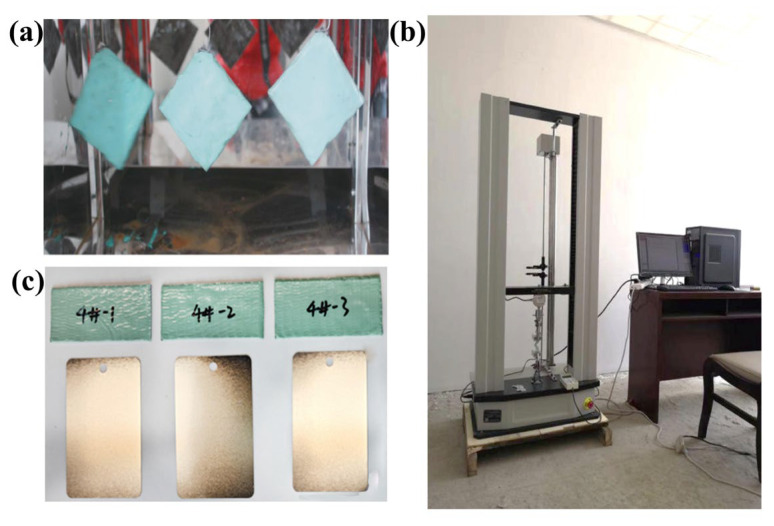
Repair testing experiments. (**a**) Drip test, (**b**) Peel strength test, and (**c**) Underwater adhesion performance test.

A drip test (GB/T51241-2017) [39] was conducted. Three samples measuring 150 mm in length and 50 mm in width were cut. After cutting, the samples were freely suspended without friction in a constant temperature oven at 85 ± 2 °C for 48 h. At the end of the experiment, the samples were removed and observed for any dripping. To test the limit performance of the internal protection materials, a subsequent experiment was conducted at 100 ± 2 °C, as shown in Figure 5a. Steel peel strength testing (GB/T2792-2014) [40] was conducted. First, the steel plate surface was wiped with acetone and allowed to dry. Three 300 mm long samples were cut from the polymer protective material samples. One end of the sample’s adhesive surface was folded back to form a folded layer of about 12 mm. Holding this folded layer, the other end was attached to one end of the steel plate, allowing the adhesive tape to suspend above the steel plate. The sample was manually rolled twice to ensure no bubbles remained. For the peel test, one end of the material was first peeled 25 mm from the steel plate. The steel plate and the free end of the material were separately clamped in the tensile testing machine fixtures, and continuous peeling was performed at a rate of (5.0 ± 0.2) mm/s. The initial 25 mm peeling data was discarded, and the average peeling force of the subsequent 50 mm was converted to peel strength (N/cm). The final result was characterized by the average of three tests, as shown in Figure 5b. Based on this, underwater adhesion performance was tested using tinplate sheets of 60 mm × 100 mm as the substrate, manually sanded to St3 grade. Three pieces of 40 mm × 80 mm 4# polymeric internal protection material samples were cut. The substrate was horizontally placed, immersed in water, and the internal protection material samples were evenly applied to the substrate and pressed. After 1 min, they were peeled off, and the retention rate of the adhesive layer was observed, as shown in Figure 5c.

### 2.4. Reinforcement System Optimization

#### 2.4.1. Reinforcement Materials

Based on the usage environment, polymeric reinforcement materials have certain flexibility and may detach or become damaged under long-term water flow scouring. Therefore, additional reinforcement materials are needed to achieve long-term protective repair effects. Single-component organosilicon reinforcement materials and two-component solvent-free epoxy reinforcement materials were designed, respectively.

**Figure 6:**
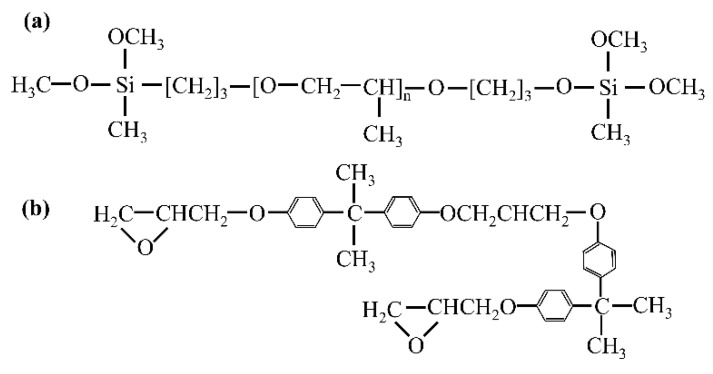
Reinforcement materials. (**a**) Modified silicone reinforcement material and (**b**) solvent-free epoxy reinforcement material.

The single-component organosilicon reinforcement material (as shown in Figure 6a) uses a moisture-curing silane-modified polyether system. Its terminal alkoxy groups undergo hydrolysis and condensation reactions with environmental moisture under catalyst action, releasing small molecule alcohols and forming a three-dimensional crosslinked network. It combines the weather resistance of organosilicon (UV aging resistance > 1000 h) with the flexibility of polyurethane (elongation at break > 300%), and can surface-dry within 30 min underwater without solvent evaporation pollution. This information was obtained from the supplier. However, its relatively low crosslinking density (Shore hardness A30-A40) results in limited resistance to mechanical damage.

The two-component solvent-free epoxy reinforcement material (as shown in Figure 6b) consists of modified epoxy resin (E-51), nano-silica fillers, and latent curing agents. The epoxy groups in its molecular chains form chemical bonds with hydroxyl groups on the substrate surface, while high-density polar groups provide physical adsorption, building high-strength interfacial bonding (pull-off strength > 3.5 MPa). This system has a curing shrinkage rate of only 0.15–0.2% (with 50 wt% filler added), significantly lower than traditional thermosetting resins (1–2%). Its glass transition temperature (Tg > 120 °C) and low coefficient of linear thermal expansion (4.5 × 10^−5^/°C) provide excellent dimensional stability, maintaining interfacial integrity during temperature cycling between −40 °C and 120 °C. The two types of materials achieve underwater rapid repair and long-term protection through different curing mechanisms, providing diversified reinforcement solutions for marine engineering structures.

Although polyurethane-based materials (e.g., the modified silane polyether system in the paper) have excellent flexibility, their hardness and abrasion resistance are relatively insufficient for offshore riser repair scenarios that require long-term resistance to seawater washout and mechanical damage. In contrast, the two-component solvent-free epoxy system ultimately selected for this study has excellent adhesion to metal substrates, lower cure shrinkage, higher hardness, and excellent resistance to chemical media. These properties enable the formation of a stronger and more durable protective layer, making it a better choice for long-term reliable protection.

#### 2.4.2. Adhesion Testing of Reinforcement Materials

**Figure 7:**
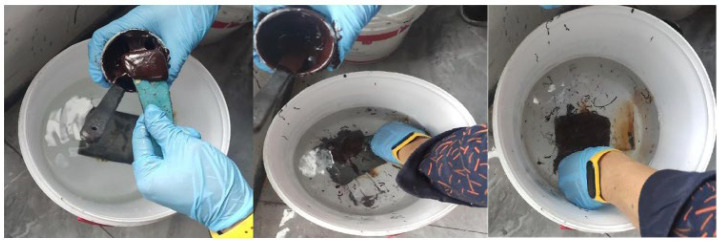
Adhesion testing of reinforcement materials.

To verify the underwater adhesion performance of reinforcement materials, simulated marine environment tests were conducted. First, 245 g of sodium chloride was dissolved in 7 kg of deionized water to prepare a 3.5 wt% sodium chloride solution to simulate the seawater environment. The two-component solvent-free epoxy reinforcement material was mixed at a mass ratio of A:B = 3:2, mechanically stirred for 5–10 min until homogeneous, and then allowed to mature for use. Six 100 mm × 100 mm tinplate sheets were selected and manually sanded to the St3 level surface treatment standard. The substrates were completely immersed in the salt solution, and the two-component epoxy material was evenly coated using a scraper (thickness controlled at 0.3–0.5 mm), with the specimens left to cure for 48 h. Simultaneously, coating tests of single-component organosilicon material were conducted in parallel, using the same process to ensure the coating layer was free of pore defects. The experimental process is shown in Figure 7.

#### 2.4.3. Performance Testing Experiments of the Reinforcement System

To systematically evaluate the engineering applicability of the two-component solvent-free epoxy reinforcement materials, key performance indicators such as adhesion, solid content, hardness, and drying time were quantitatively characterized to provide a scientific basis for material design optimization and engineering applications. The pull-off method was used to test the adhesion of underwater-cured coatings. The AB adhesive with a 1:1 ratio was evenly coated on the surface of the cured iron plate, and after attaching dollies, it was left to cure. An adhesion tester (GB/T 5210-2006 [41]) was used to apply a pulling force in the vertical direction. The solid content of the material was determined by the weight loss method. Three groups of dried weighing bottles (*M*_1_) were taken, 1 g of uniformly mixed AB components (*M*) was added to each, dried at 105 °C for 3 h, and then cooled and weighed (*M*_2_). Calculation was performed according to Formula (4).

X = (*M*_2_ − *M*_1_)/*M* × 100% (4)

**Figure 8:**
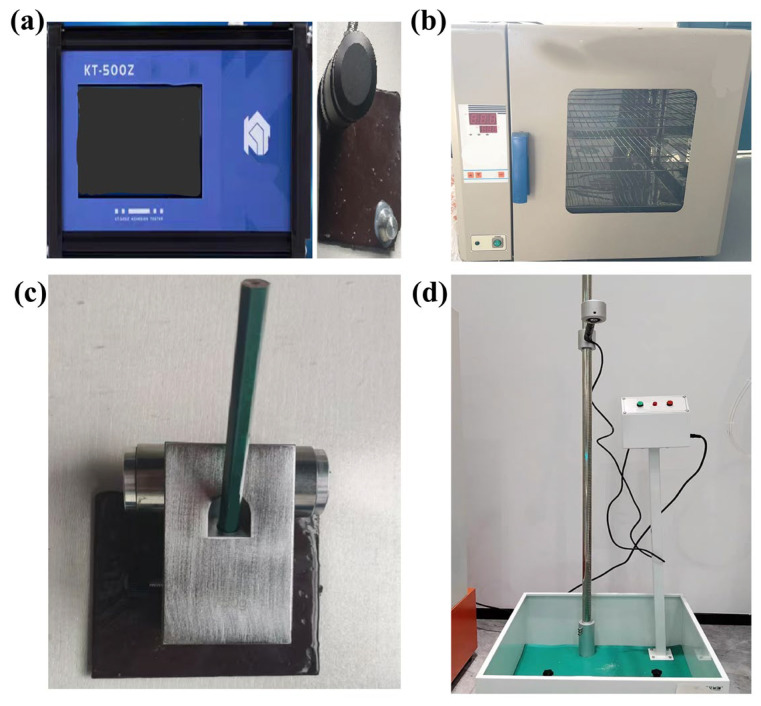
Performance testing experiments of the reinforcement system. (**a**) Adhesion test experiment, (**b**)Solids test, (**c**) Pencil hardness rating test, and (**d**)Impact resistance test.

The mechanical pencil sharpening method (GB/T 6739-2022 [42]) was used to prepare standard pencil leads, which were drawn at a 90° perpendicular angle on the coating surface at a constant speed. The hardness grade was determined by observing scratches through a magnifying glass. An impact test stand (GB/T 1732-2020 [43]) was used to test impact resistance. The drying characteristics (GB/T 9272-1988 [44]) were jointly evaluated using the finger-touch method and cotton ball method. Surface drying was defined as the coating film not sticking to hands but slightly tacky, while actual drying was judged by no residual marks from a cotton ball. The experimental process and specific equipment are shown in Figure 8 and Table 2.

### 2.5. Application Experiment on Offshore Oil and Gas Well Conductor

**Figure 9:**
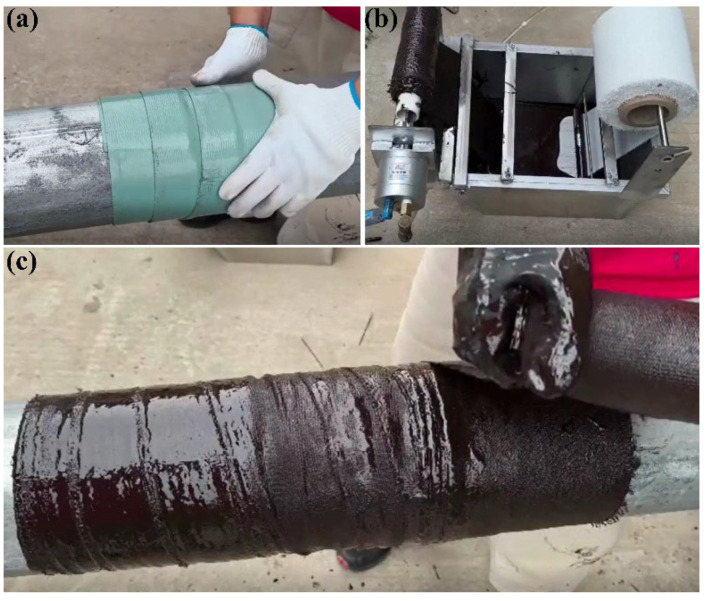
Application experiment on offshore oil and gas well conductor. (**a**) Internal protection material, (**b**) Coated high-strength fiber tape, and (**c**) Wrapped nano-polymer epoxy material.

To verify the protective performance of the overall material on offshore oil and gas well conductors, suitable repair locations were first selected, and the casing base was cleaned to remove impurities such as oil stains and rust, ensuring a clean construction interface and creating conditions for material adhesion. Subsequently, internal protection polymer material was wrapped, as shown in Figure 9a, to build a basic protective layer and enhance the corrosion resistance of the conductor. Next, high-strength fiber tape was applied, as shown in Figure 9b, to enhance structural strength to resist external impacts in the marine environment. Nano-polymer epoxy material was then wrapped, as shown in Figure 9c, to further optimize sealing and protection capabilities to adapt to complex sea conditions. Finally, curing inspection was conducted to verify the curing effect of each layer of material, ensuring reliable protective structure and stable resistance of the conductor to marine corrosion, mechanical loads, and other challenges during long-term use, maintaining normal operation of oil and gas wells. The experimental process is shown in Figure 9.

## 3. Results and Discussion

### 3.1. Results and Discussion of Material Characterization Verification Experiments

#### 3.1.1. Basic Performance Experiments

**Figure 10:**
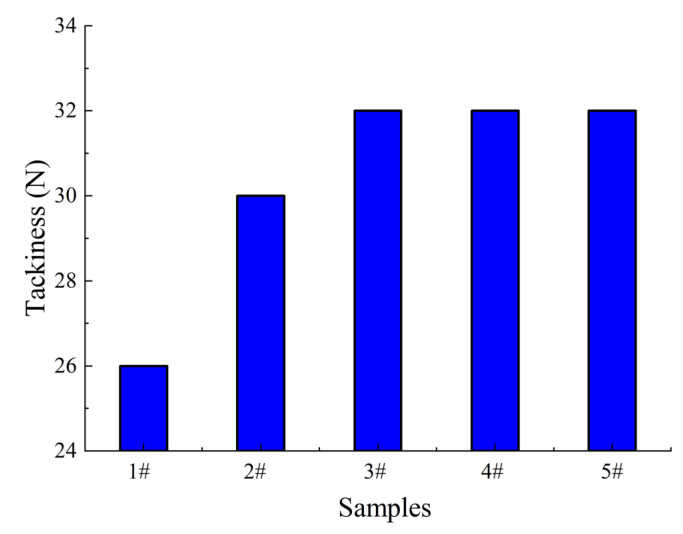
Differences in adhesive tackiness among different samples.

Figure 10 shows the differences in adhesive tackiness among different samples. Observations show that from samples 1# to 5#, the adhesive tackiness exhibits a gradually increasing trend, with 1# having the lowest tackiness (26 N), while 3#, 4#, and 5# all reach relatively high levels (around 32 N). Combining image and formula analysis to understand the mechanism: as the proportion of PIB in the matrix increases, the double bond content in the matrix decreases, and PIB itself has a lower molecular weight than butyl rubber, resulting in a lower modulus. These two major factors work together to significantly increase the surface energy and tackiness of the adhesive. Specifically, the increased proportion of PIB weakens the inhibitory effect of double bonds on tackiness in the system, and combined with its low modulus characteristics, further reduces the mechanical resistance within the adhesive that hinders tackiness formation, ultimately promoting enhanced surface tackiness of the adhesive. This pattern indicates that the PIB ratio is a key factor in regulating adhesive tackiness, and adjusting the amount of PIB can effectively optimize the surface adhesion performance of the adhesive, providing an important basis for the design of relevant material formulations.

**Figure 11:**
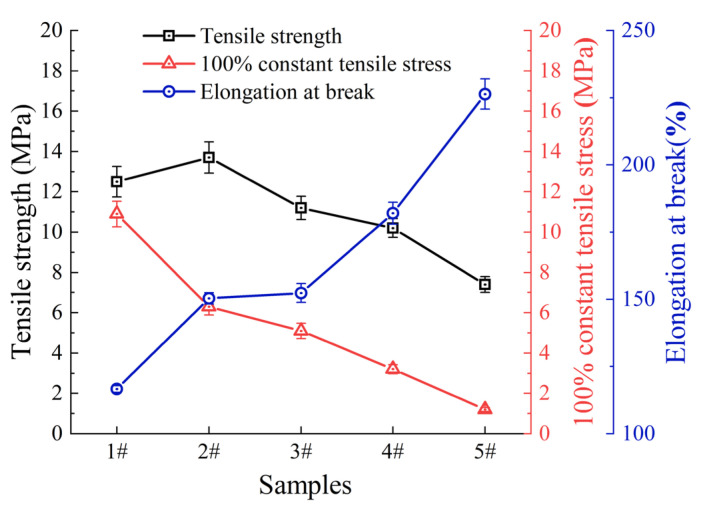
Experimental results of different amounts of PIB.

Figure 11 shows the effects of different amounts of PIB on the tensile strength, 100% modulus, and elongation at break of the blend system. Analysis of the Figure 11 reveals that as the amount of PIB increases, the 100% modulus of the blend system shows a significant decreasing trend, with a maximum decrease of 42% compared to the sample without PIB (formulation 1#), indicating that increasing PIB content significantly weakens the stiffness of the system. The elongation at break shows an inverse change, significantly increasing with the increase of PIB content, with a maximum increase of 24%, indicating that PIB can effectively enhance the extensibility of the system. The trend of tensile strength change is rather unique, showing a pattern of initial increase followed by decrease with increasing PIB content, with a maximum decrease of 18%, suggesting that there exists a critical value of PIB content that affects tensile strength, beyond which the tensile strength of the system will weaken. The significant increase in elongation at break is mainly attributed to the internal plasticizing effect of the m-PIB molecular chains, which increases the mobility of the IIR network chain segments and, thus, enhances the toughness of the system. The tendency of the tensile strength to increase and then decrease reflects an equilibrium: the introduction of a small amount of PIB may improve the interfacial compatibility of the two phases, which contributes to the stress transfer and energy dissipation and, thus, improves the strength; however, when the content of PIB is too high, the non-crosslinked and lower-strength PIB phase becomes the “weak point” in the system, which disrupts the continuity of the IIR continuity of the crosslinked network, leading to a decrease in overall tensile strength. Overall, the amount of PIB significantly affects the mechanical properties of the blend system, with different performance indicators showing differentiated patterns with changes in PIB content, providing data reference for formula optimization of the blend system.

Isobutylene itself is one of the structural components of IIR, with good compatibility with IIR. Through the action of mechanical shear forces, PIB and IIR can effectively form a homogeneous and stable blend. It is proposed that PIB macromolecules randomly penetrate through the IIR crosslinked network, which is believed to improve the processing performance, as qualitatively evidenced by the successful material preparation via kneading and extrusion described in Section 2.2. Comprehensive comparison shows that Formula 4# has good initial tackiness, and better comprehensive performance in terms of tensile strength and modulus. Formula 4# was ultimately selected for trial production and related testing.

#### 3.1.2. Repair Test Results

**Figure 12:**
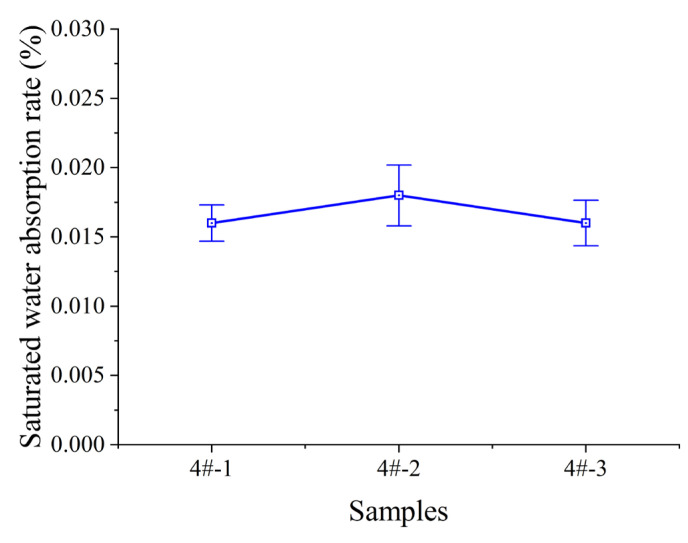
Saturated water absorption rates of three samples.

As shown in Figure 12, the saturated water absorption rates of three tested samples all meet the requirement of ≤0.03%, indicating that the samples have excellent water barrier properties and can effectively block the penetration of corrosive media such as seawater when used for underwater wrapping in marine environments, providing excellent protection. Additionally, in the drip test, the samples could meet the requirement of no dripping at >80 °C, and could achieve no dripping at temperatures up to 100 °C.

**Figure 13:**
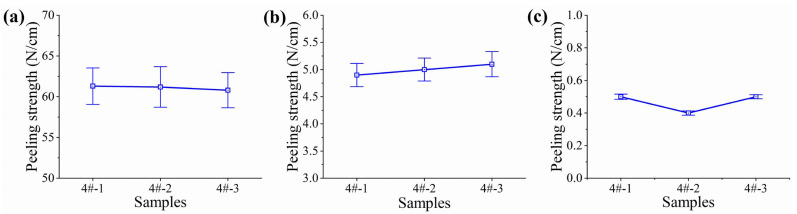
Peel strength against steel test results. (**a**) −45 °C, (**b**) 23 °C, and (**c**) 80 °C.

As shown in Figure 13, as the temperature gradually increases from low to room temperature and then to high temperature, the peel strength against steel of three test strips of Formula 4# internal protection material all show a decreasing trend. Despite the strength changes affected by temperature, under various test temperature conditions of low, room, and high temperatures, the peel strength against steel of the Formula 4# internal protection material samples always remains greater than 0.3 N/cm. The peel strength against steel of the Formula 4# internal protection material samples always remains greater than the target value of 0.3 N/cm. This demonstrates the stable performance of the material under different temperature ranges.

**Figure 14:**
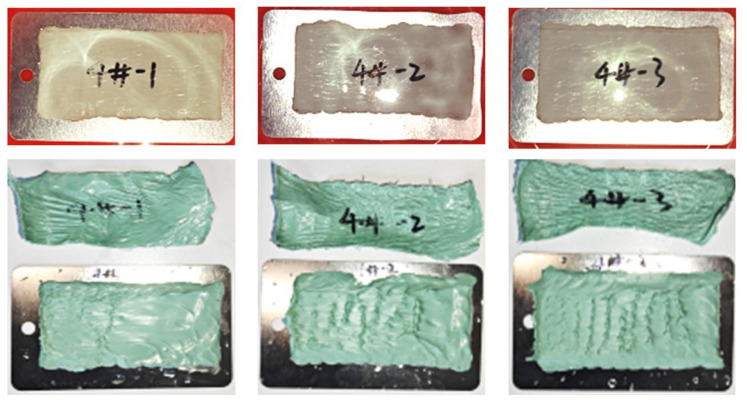
Underwater adhesion performance test results of internal protection material samples.

As shown in Figure 14, the underwater adhesion performance test results of the internal protection material samples are displayed, with all three samples having adhesive layer retention rates >95%. This indicates that the internal protection material can achieve good adhesion with the substrate underwater, with excellent adhesion strength.

### 3.2. Results and Discussion of Reinforcement System Performance Testing Experiments

Single-component organosilicon reinforcement materials, due to their flexible chain segments, still have insufficient hardness after curing, even after modification, and cannot achieve the desired effect. Two-component solvent-free epoxy has high strength after curing, with many epoxy active groups and densely entangled chain segments, which can effectively reinforce polymer reinforcement materials. The performance of solvent-free epoxy reinforcement material is shown in Table 3.

Through a series of research and testing, this polymer protection and repair material system can not only provide wrapper protection onshore, serve as an anti-corrosion layer for in-service pipeline anti-corrosion layer repair and new pipeline joint patching, providing good sealing properties and effectively preventing the invasion of various chemical substances, but can also be implemented underwater, particularly suitable for preventive wrapping protection and leakage sealing repair protection of wellbores.

### 3.3. Results and Discussion of Application Experiments on Offshore Oil and Gas Well Conductors

**Figure 15:**
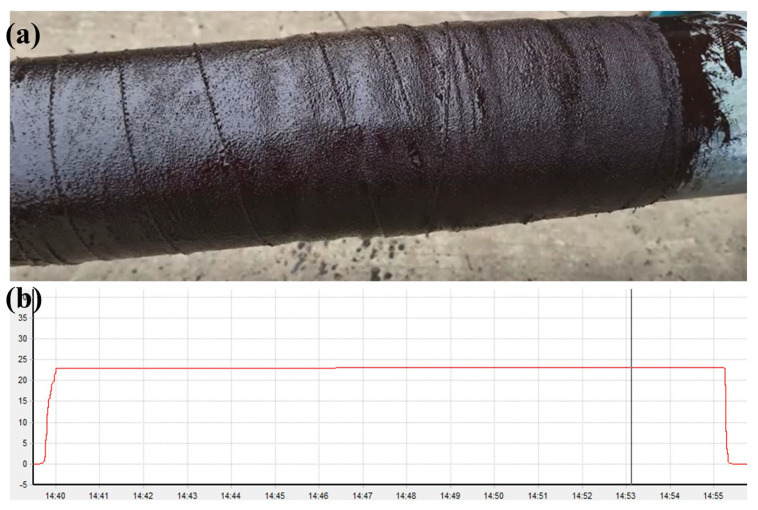
Application experiments on offshore oil and gas well conductors. (**a**) Curing effect, (**b**) Pressure retention results.

This experiment aims to explore the application performance of the combination of internal protection polymer material and two-component solvent-free epoxy. From Figure 15a, it can be clearly seen that after the combined action of the two materials, the curing effect is very satisfactory. After a 24-h curing cycle, the formed cured material closely adheres to the inner wall of the pipe, effectively achieving pipe sealing and preventing medium leakage.

To test the stability of the material under complex working conditions, internal high-pressure tests were conducted. As can be seen from Figure 15b, when applying 25 MPa pressure to the pipe and maintaining the pressure for 15 min, the pressure remained constant throughout, with no separation between the material and the pipe, and no pressure fluctuation, demonstrating good pressure resistance stability. In addition, the material peel strength test shows that the lower the temperature, the higher the peel strength, maintaining firmness even in low-temperature environments. Impact resistance tests indicate that, against the impact force generated by long-term scouring of seawater, the material can effectively protect the damaged areas of the pipe, ensuring the integrity of the pipe structure and function.

## 4. Conclusions

This research focuses on the corrosion protection challenge of marine petroleum conductors, and based on a polyisobutylene (PIB) and butyl rubber (IIR) blend system, successfully developed a new underwater curing composite material, achieving a series of key results:

(1) The internal plasticizing effect of medium molecular weight PIB synergizes with the IIR crosslinking network, breaking the contradiction between material elasticity and strength; utilizing the hypothesized interfacial effect of nano-fillers, a stable peel strength of ≥0.3 N/cm is achieved across a wide temperature range from −45 °C to 80 °C. This mechanism warrants further microstructural investigation in future work.
(2) Innovative two-component solvent-free epoxy system, combining medium molecular weight PIB internal plasticization, nano-silica hydrogen bond enhancement, and latent curing agent regulation, achieving rapid surface drying in 30 min underwater and pull-off strength > 3.5 MPa.
(3) Formula 4# has the best comprehensive performance, with peel strength against steel > 0.3 N/cm at different temperature ranges, and underwater adhesive layer retention rate > 95%. The two-component solvent-free epoxy reinforcement material has high strength after curing, can effectively reinforce polymer materials, and meets engineering requirements.
(4) The combined action of internal protection polymer material and two-component solvent-free epoxy can seal pipes after 24 h of curing. Under internal high-pressure tests, there are no abnormalities with 25 MPa pressure maintained for 15 min, and the material has high peel strength at low temperatures, is impact-resistant, and can protect damaged areas of pipes.

This research provides theoretical and technical support for the repair process of marine petroleum conductors, and the developed materials have broad application prospects in the field of marine engineering protection. In the future, in the marine petroleum industry, such composite materials will help offshore oil and gas exploitation advance towards deep and remote seas. From conductor installation to platform protection, with excellent performance, it can not only reduce maintenance costs and extend equipment service life, but also ensure the safety of exploitation operations. The implementation of ISO standards will also promote its widespread application in the global marine petroleum market.

## Figures and Tables

**Table 1:** Polymer internal protection material codes and synergistic composition ratios (parts by weight).

Parts by Weight	1#	2#	3#	4#	5#
	Number of Copies				
IIR	240	192	168	144	120
PIB	0	48	72	96	120

**Table 2:** Types of equipment used in the experiment and contents of the experiment.

Equipment	Contents of the Experiment (Brand and Model Name, Manufacturers and Locations, Test Details)
Single-screw extruder	JYM SJ 30, (qdjingke Plastic Machinery Co., Ltd., Qingdao, Shandong Province, China, prepared composite samples with fiber-reinforced mesh sandwiched between them)
Universal testing machine	WDW i5, (Shandong Shijin Instrument Equipment Co., Ltd., Jinan, Shandong Province, China; HST UTM5305, Jinan Hengsi Shengda Instrument Co., Ltd. Jinan, Shandong Province, China; initial adhesion test and underwater adhesion performance test)
Adhesion tester	KT-500Z, (Shenzhen KeTan Electronic Technology Co., Ltd., Shenzhen, Guangdong Province, China, adhesion test experiment (100 × 100 mm, apply thickness of 0.3–0.5 mm))
Thermal oven	KV 101-2A, (Shandong Yisheng Heavy Industry Technology Co., Ltd., Taian, Shandong Province, China, drying test (100 × 100, apply thickness of 0.3–0.5 mm))
Pencil hardness rating tester	OU4300-2H, (Cangzhou Oupu Testing Instrument Co., Ltd., Cangzhou, Hebei Province, China, determination of coating surface hardness (100 × 100, apply thickness of 0.3–0.5 mm))
Impact tester	BF-F-315ST, (Guangdong Bell Experiment Equipment Co., Ltd., Dongguan, Guangdong Province, China, determination of the overall hardness of the sample via the free-fall experiment (100 × 100, apply thickness of 0.3–0.5 mm))

**Table 3:** Performance of solvent-free epoxy reinforcement material.

Item	Technical Specifications
Color	Dark red
Solid content, %	99.3
Adhesion, MPa	3
Impact resistance, cm	50
Drying time, h	Surface dry ≤ 1.5
	Thoroughly dry ≤ 24
Pencil hardness	6H
Temperature resistance	120 °C
Salt spray resistance	No abnormality after 1000 h

## Data Availability

The original contributions presented in this study are included in the article. Further inquiries can be directed to the corresponding author(s).

## Abbreviations

The following abbreviations are used in this manuscript:

IIR	Butyl rubber
DOP	Dioctyl phthalate
m-PIB	Medium molecular weight polyisobutylene
PIB	Polyisobutylene
